# A Novel CD206 Targeting Peptide Inhibits Bleomycin-Induced Pulmonary Fibrosis in Mice

**DOI:** 10.3390/cells12091254

**Published:** 2023-04-26

**Authors:** Anghesom Ghebremedhin, Ahmad Bin Salam, Benjamin Adu-Addai, Steve Noonan, Richard Stratton, Md Shakir Uddin Ahmed, Chandra Khantwal, George R. Martin, Huixian Lin, Chris Andrews, Balasubramanyam Karanam, Udo Rudloff, Henry Lopez, Jesse Jaynes, Clayton Yates

**Affiliations:** 1 Moores Cancer Center, University of California San Diego, La Jolla, CA 92093, USA; 2 Department of Biology and Center for Cancer Research, Tuskegee University, Carver Research Foundation, Tuskegee, AL 36088, USA; 3 Department of Pathobiology, College of Veterinary Medicine, Tuskegee University, Tuskegee, AL 36088, USA; 4 Murigenics Inc., 941 Railroad Ave., Vallejo, CA 94592, USA; 5 Royal Free Hospital, UCL Division of Medicine, University College London, London WC1E 6JF, UK; 6 Bangladesh Council of Scientific and Industrial Research, Dhaka 1205, Bangladesh; 7 Riptide Bioscience, 941 Railroad Ave., Vallejo, CA 94592, USA; 8 Rare Tumor Initiative, Pediatric Oncology Branch, Center for Cancer Research, National Cancer Institute, Bethesda, MD 20892, USA; 9 College of Agriculture, Environment and Nutrition Sciences, Tuskegee University, Tuskegee, AL 36088, USA

**Keywords:** macrophages, myofibroblasts, IPF, immunotherapy, CD206

## Abstract

Activated M2-polarized macrophages are drivers of pulmonary fibrosis in several clinical scenarios, including Idiopathic Pulmonary Fibrosis (IPF). In this study, we investigated the effects of targeting the CD206 receptor in M2-like macrophages with a novel synthetic analogue of a naturally occurring Host Defense Peptide (HDP), RP-832c, to decrease profibrotic cytokines. RP-832c selectively binds to CD206 on M2-polarized bone marrow-derived macrophages (BMDM) in vitro, resulting in a time-dependent decrease in CD206 expression and a transient increase in M1-macrophage marker TNF-α. To elucidate the antifibrotic effects of RP-832c, we used a murine model of bleomycin (BLM)-induced early-stage pulmonary fibrosis. RP-832c significantly reduced fibrosis in a dose-dependent manner, and decreased CD206, TGF-β1, and α-SMA expression in mouse lungs. Similarly, in an established model of lung fibrosis, RP-832c significantly decreased lung fibrosis and significantly decreased inflammatory cytokines TNF-α, IL-6, IL-10, IFN-γ, CXCL1/2, and fibrosis markers TGF-β1 and MMP-13. In comparison with the FDA-approved drugs Nintedanib and Pirfenidone, RP-832c exhibited a similar reduction in fibrosis compared to Pirfenidone, and to a greater extent than Nintedanib, with no apparent toxicities observed. In summary, our findings showed that inhibiting the profibrotic alternatively activated M2-like macrophages using a novel peptide, RP-832c, could reduce BLM-induced pulmonary fibrosis in mice, warranting the therapeutic potential of this peptide for patients with pulmonary fibrosis.

## 1. Introduction

Multiple fibrotic diseases such as acute lung injury (ALI), scleroderma, and idiopathic pulmonary fibrosis (IPF) share many pathophysiological features, including a pro-inflammatory stimulus leading to a rapid release of IL-8 and IL-6 by alveolar macrophages that further attract neutrophils, causing alveolar and endothelial injury [1] and ultimately resulting in high mortality rates. Although the majority of IPF patients experience a relatively slow disease progression, there is a subset of rapid progressors that demonstrate the upregulation of inflammatory pathways and have an accelerated loss of lung function and shorter survival [2,3]. The poor survival of rapidly progressing IPF patients is directly attributed to the deposition of dense parenchymal fibrosis, resulting in an ultimate loss of pulmonary function. Thus, there is a great need for therapeutics that block the fibrotic features of these conditions. Increased myofibroblasts, deposition of collagen, and alveolar epithelial injury are characteristics of IPF and bleomycin-induced lung fibrosis in animals, resulting in impaired functional gas exchange, respiratory failure, and even death [4,5,6,7].

Macrophages are the most abundant innate immune cells in the lung, and they play an important role in the pathogenesis of pulmonary fibrosis [8,9]. The reciprocal interaction between fibroblasts and macrophages in the lung prominently drives the progression of most fibrotic illnesses. Irrespective of how they are recruited into areas of tissue injury, pro-fibrotic macrophages commonly coordinate scar formation through a range of interactions with myofibroblasts, which are the main cellular source of pathological ECM deposition during fibrosis [10,11,12,13,14,15].

It has been suggested that the polarization of alveolar macrophages toward a profibrotic or “M2” phenotype contributes to the development of fibrosis [16,17]. These macrophages regulate fibrosis by secreting growth factors and cytokines, including TGF-β1, that recruit and activate fibroblasts and other inflammatory cells [18], which in turn promote collagen-producing myofibroblasts [19,20,21]. Minutti, C. M. et al. have recently shown that macrophage-derived amphiregulin induced the differentiation of mesenchymal stromal cells into myofibroblasts via integrin-αV-mediated activation of TGF-β1 [22]. Macrophages can exhibit various phenotypes, with M1 classically activated macrophages classified using canonical markers such as IFN-γ, CD80, and CD86. The M2 alternatively activated macrophages show a high expression of CD163 and CD206 receptors and induce immune suppression [21]. Several studies have demonstrated that activated CD206-positive M2 macrophages in fibrotic lesions produce high amounts of IL-10, IL-6, TNF-α, and TGF-β1 that enhance collagen synthesis and deposition [15,23]. These pro-inflammatory and profibrotic cytokines produced by M2 macrophages indirectly inhibit the production of anti-inflammatory cytokines in a negative feedback loop, further promoting fibrosis [24,25,26].

Host Defense Peptides (HDP) are ubiquitously expressed in many complex organisms and are critical mediators of the innate immune response [27]. Recently, several groups have identified fragments of these HDPs (10–15 aa.) which have immunomodulatory activity [27,28]. These 10–15 aa fragments were also found in internal sequences of collagens, complements, and virulence factors, including pathogens such as bacteria and viruses that induce cellular changes in many immune cell types including leukocytes and macrophages [27,29]. Our group reported that HDPs, RP-182, and RP-832c, specifically target the CD206 receptor on M2 macrophages, inducing a major conformation change that activates a signaling pathway that rapidly induces apoptosis in CD206-positive M2 macrophages and repolarization toward the M1 phenotype [29]. Although the efficacy of these peptides has been extensively characterized in multiple tumor models, it has not yet been well characterized in lung fibrosis. The bleomycin (BLM)-induced model of inflammation and fibrosis represents an experimental model for lung fibrosis [30,31]; therefore, we used this model to define the role of RP-832c more completely in lung fibrosis.

## 2. Materials and Methods

The RP-832c (RWKFGGFKWR) peptide was synthesized by PolyPeptide Laboratories, San Diego, CA, USA. BMDM cells were isolated and polarized as described previously [31]. The cell viability of RP-832c-treated macrophages was determined using the live/dead viability assay as previously described [29]. Human fetal lung fibroblast cell lines MRC5, IMR90, and IMR9 were purchased from the American Type Culture Collection (ATCC, Manassas, VA, USA) and were cultured according to ATCC protocols.

### 2.1. Cell Culture of Primary Cells

BMDM cells were isolated as described previously [32]. Briefly, murine monocyte precursor cells were obtained by flushing out the bone marrow from the femur bones of 6–8-week-old healthy C57BL/6 mice. These precursor cells were then differentiated into M1 macrophages using 20 ng/mL of M-CSF and 20 ng/mL of IFN-γ and into M2 macrophages using 20 ng/mL of M-CSF and 20 ng/mL of IL-4 [29]. Human fetal lung fibroblast cell lines MRC5, IMR90, and IMR9 were purchased from the American Type Culture Collection (ATCC, Manassas, VA, USA) and were cultured according to ATCC protocols.

### 2.2. Cell Viability Assay

Cell viability dose-response curves were determined using the Live/Dead Viability/Cytotoxicity Kit (Thermo Fisher Scientific, Waltham, MA, USA, Cat# L3224) using the manufacturer’s protocol. Macrophages were seeded onto glass-bottom 96-well plates and polarized into M1 or M2 populations. Cells were treated with different concentrations of RP-832c for 48 h. After drug treatment, a 100 μL mixture of 2 μM calcein-AM and 4 μM ethidium homodimer was added and incubated for 1 h. The images were taken and analyzed as described in the immunofluorescence assay section below. A total of 200 cells were counted manually from three different random 20× regions across three technical replicates, and the percentage of alive cells was calculated using GraphPad Prism version 8.0.

### 2.3. Animal Experiments

C57BL/6J mice were obtained from Envigo, Indianapolis, IN, USA. All animal studies were approved by the IACUC (Murigenics, Inc., Vallejo, CA, USA). After 3 days of acclimation, the mice were challenged with a single 2.5 U/kg body weight dose of bleomycin (BLM) intratracheally (IT), and a vehicle (normal saline) or RP-832c(QD) was administered subcutaneously for 21 days starting 3- and 14-days post-BLM-challenge. Body weights were measured daily, and lung weight was measured at the end of each study.

### 2.4. Histological and Immunostaining Evaluation

At the end of the study, the mice were sacrificed using CO_2_ followed by cervical dislocation (according to IACUC guidelines), and the lungs were collected and fixed in 10% formalin. After 48 h of fixation, the lungs were processed into tissue sections. The tissue sections were stained with both Hematoxylin/Eosin (H&E) (Sigma Aldrich, Burlington, MA, USA) and Masson Trichrome (Abcam, Waltham, MA, USA). Immunostaining was performed with the anti-CD206 (AF2535, 5 ug/mL, R&D Biosciences), TGF-β1 (MAB1835, 10 ug/mL, R&D Biosciences), and α-SMA (MAB1420, 10 ug/mL, R&D Biosciences, Minneapolis, MN, USA) antibodies as previously described [29]. All slides were scanned using a Leica Aperio SC2 scanner and blindly evaluated by two independent pathologists. A total of 10 representative fields of 20× IHC images per tissue section were individually analyzed for the total number of cells/area using a confocal microscope (Olympus, New York, NY, USA). All quantitative data were normalized to appropriate control images.

### 2.5. Immunofluorescence

Tissues were fixed with cold 100% methanol alone, permeabilized with 100 mM Tris–HCl (pH 7.4), 150 mM NaCl; 10 mM EGTA; 1% Triton X-100; 1 mM PMSF; and 50 μg/mL aprotinin (all from Sigma) for 30 min, and subsequently blocked with 5% bovine serum albumin for 1 h at room temperature. Samples were incubated with the indicated primary antibodies diluted in a blocking buffer at 4 °C overnight. The samples were then incubated with secondary antibodies (Invitrogen’s Alexa Fluor 488 and 594 anti-mouse and anti-rabbit antibodies) for 1 h at room temperature. Nuclear staining was accomplished with Vectashield mounting medium containing DAPI, and staining was observed with a DSU confocal microscope (Olympus, New York, NY, USA). Quantifications of immunofluorescence staining were carried out by measuring the mean fluorescence intensity of 10 different 20× images of each tissue using Metamorph Imaging Software (Molecular Devices, LLC, Sunnyvale, CA, USA). Bar graphs represent an average mean intensity or number of cells of the lung tissue sections. All quantitative data were normalized to appropriate control images.

### 2.6. Quantitative Real-Time PCR

The quantification of individual mRNA expression was performed by real-time PCR on an ABI 7500 Fast Real-Time System (Applied Biosystems, Foster City, CA, USA) using TaqMan probes as previously described [25]. Total RNA was extracted from each cell type or FFPE (Formalin-Fixed Paraffin-Embedded) tissue section using RNAzol (Molecular Research Center, Inc., Cincinnati, OH, USA) according to the manufacturer’s protocol. RNA (1000 ng) was reverse-transcribed using TaqMan mRNA reverse transcription kits (Life Technologies, Carlsbad, CA, USA). The relative expression of mRNAs was quantified with the TaqMan Universal PCR Master Mix, No AmpErase UNG, using the One-Step Real-Time PCR system (Life Technologies). Thermal cycling conditions included enzyme activation for 10 min at 95 °C, 40 cycles at 95 °C for 15 s, and 60 °C for 60 s. The following TaqMan probes were used: IL-10 (Mm00439615_g1), CXCL-2(Mm00436450_m1), CXCL-1(Mm04207460_m1), TGF-β1 (Mm00441727_g1), IL-6(Mm00446190_m1), IFN-γ(Mm00801778_m1), IL1B (Mm00434228_m1), TNF-α,(Mm00443258_m1), MMP-13(Mm00439491_m1), CD206 (Mm01329362_m1), CD86(Mm00444540_m1), iNOS (Mm00440502_m1), and GAPDH (Mm99999915_g1). The relative expression of individual genes was determined by normalizing to the housekeeping gene GAPDH.

### 2.7. Statistical Analysis

Data are reported as means ± SD. Comparisons between the groups were performed using non-paired and non-parametric Mann-Whitney U tests. All statistical analyses were conducted using GraphPad Prism software version 8. A *p* value of <0.05 was considered statistically significant.

## 3. Results

### 3.1. RP-832c Targets CD206 Positive Macrophages

**Figure 1:**
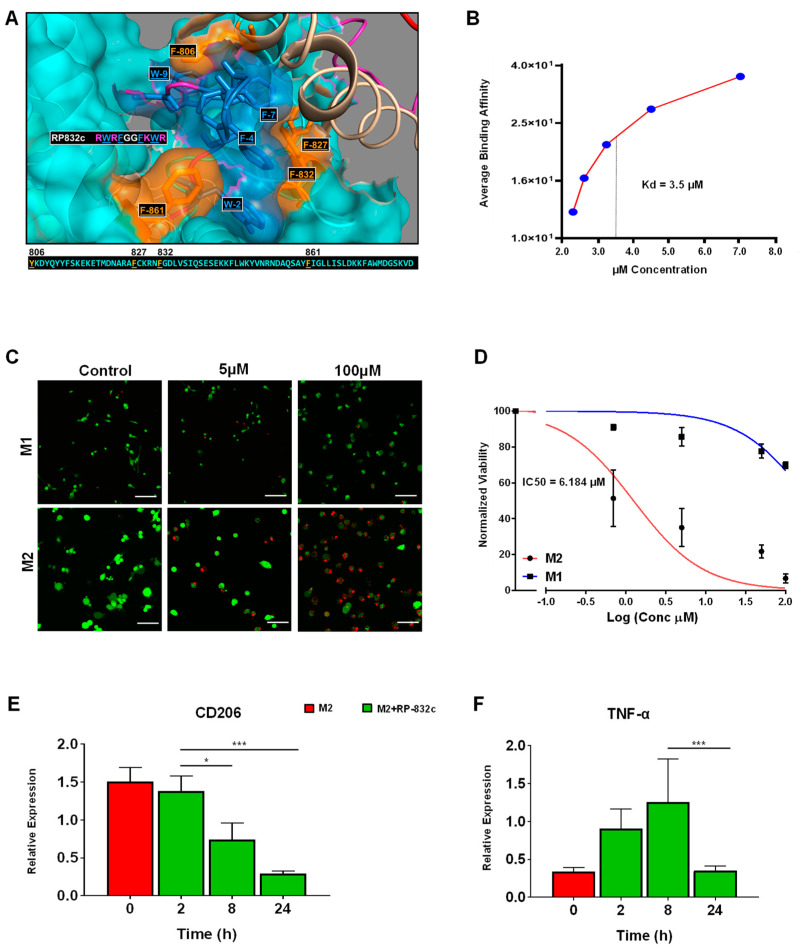
RP-832c specifically targets the CD206 receptor with high affinity. (**A**) In silico docking of the RP-832c peptide to the CD206 molecule demonstrates that the peptide binds to CD206 with a binding energy of −1349.2 kcal/mol. (**B**) Surface Plasmon Resonance (SPR) spectroscopy analysis for the binding of CD206 proteins. (**C**) Representative 20× immunofluorescence (IF) images of Live/Dead assays of M1 and M2 BMDMs after 48 h of treatment with 0–100 μM of RP-832c. Dead cells are shown in red and live cells are shown in green. (**D**) Dose–response curve of M1 and M2 BMDMs treated with 0–100 μM of RP-832c. (**E**) RT-PCR for CD206 gene expression of M2-polarized BMDM after 0–24 h of treatment with 10 μM RP-832c. (**F**) RT-PCR for TNF-α gene expression of M2-polarized BMDM after 0–24 h of treatment with 10 μM RP-832c. All data are presented as the means of three independent experiments performed in triplicate ± S. E. *** *p* < 0.0001, and * *p* < 0.05 are considered significant.

Because RP-182 is well-characterized, we sought to further characterize the physical and functional properties of RP-832c. RP-832c is an amphipathic β-sheet of two palindromic pentamers, and is predicted to bind phenylalanine residues (821, 831, and 861) in the CRD5 (C-type carbohydrate recognition domain 5) binding region of CD206 (Figure 1A). In silico docking studies predicted RP-832c to have a higher affinity/COB to full-length CD206 compared to RP-182. Surface plasma resonance (SPR) binding analysis confirmed that RP-832c binds recombinant CD206 receptor proteins rapidly in a dosage-dependent manner (Supplementary Figure S1A), with an estimated K_D_ of 3.5 µM (Figure S1B).

To determine the specificity of RP-832c for M2 macrophages, we performed live/dead assays using bone marrow-derived macrophages polarized to either M1 or M2 phenotypes (Supplementary Figure S1B). RP-832c exhibited a dosage-dependent inhibition of cell viability in M2 macrophages with an IC50 of ~6 µM, producing only minimal cytotoxicity in M1 polarized macrophages (Figure 1C,D) while lacking any cytotoxicity in two different human fetal lung fibroblast cell lines MRC5 and IMR9 when treated with up to 100 µM of RP-832c (Supplementary Figure S2). RP-832c further decreased CD206 gene expression of M2 macrophages (Figure 1E), resulting in an initial increase in TNF-α gene expression, which declined over 24 h (Figure 1F). These results suggest that RP-832c activity on macrophages is highly specific to the M2 subpopulation.

### 3.2. RP-832c Prevented Fibrosis in an Early Model of BLM-Induced Lung Fibrosis

We first sought to perform in silico validation of CD206 expression at various time points in both bleomycin-treated rat (GSE48455) and mouse models (GSE40151). Overall, CD206 expression was significantly upregulated (*p*-value < 0.0001) as early as day 2 following the BLM challenge, peaked at day 7 (*p*-value < 0.0001), and remained elevated through day 35 (Supplementary Figure S3A,B). We further validated these findings by immunohistochemistry staining and found that CD206 expression was upregulated in the BLM-treated mice (Supplementary Figure S3C,D). Thus, the BLM model appears to be an appropriate model to determine the role of CD206 in lung fibrosis.

**Figure 2:**
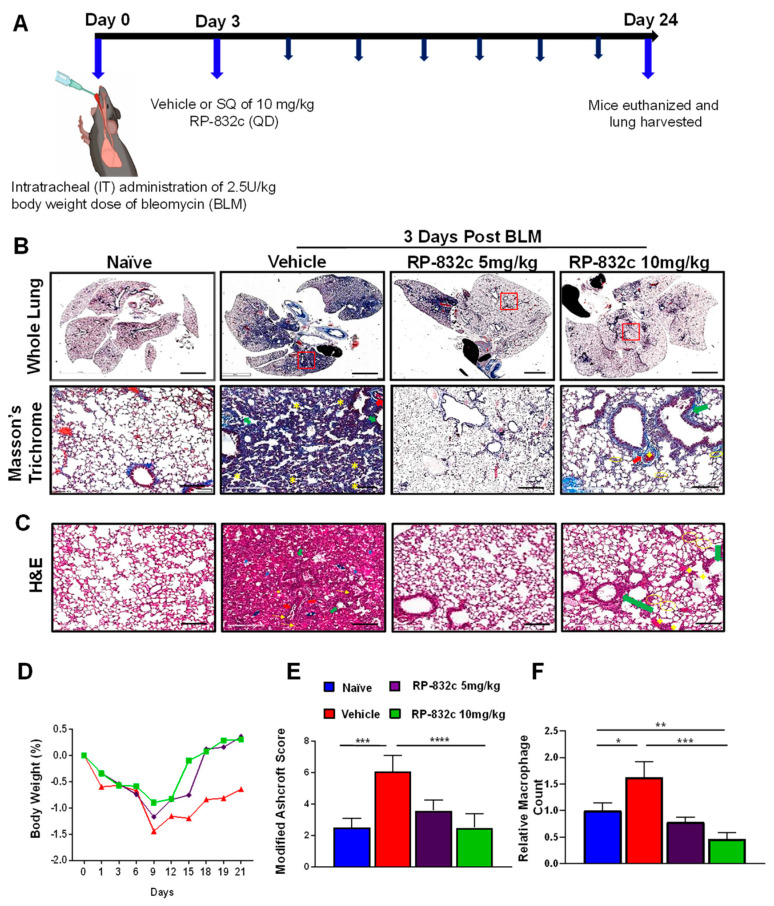
RP-832c peptide prevents fibrosis in an early model of BLM-induced lung injury. (**A**) A schematic of the animal study in which mice were challenged with 2.5 U/kg body weight doses of BLM at day 0. Three days later, the mice were treated with either vehicle, 5 mg/kg or 10 mg/kg RP-832c (QD), for an additional 21 days. (**B**) The upper panel is representative of Masson’s Trichrome-stained 2× images of whole lung tissue, and the lower panel shows representative 20× Masson’s Trichrome-stained images of the lungs. (**C**) Representative 20× images of H&E-stained lung tissue sections showing thickening of the bronchiolar wall and distended blood vessels with high cellularity. The blue arrows indicate alveolar spaces with edema and the yellow arrows indicate inflammatory cells (lymphocytes, plasma cells, and macrophages). Double arrow ends (Black) indicates some of the distorted and remaining alveolar spaces (air bubbles). Thick arrows (red) indicate areas with perivascular fibrosis. Asterisks show interstitial fibrosis/collagen. Thick arrows (green) indicate areas with parabronchial fibrosis. (**D**) The body weight of the mice in each treatment group was measured throughout the study. (**E**) Lung tissue fibrosis was assessed using the modified Ashcroft scoring system. (**F**) The total number of macrophages counted microscopically over 10 high fields of 20× H&E images. *n* = 6 mice per treatment group. S. E. **** *p* < 0.0001, *** *p* < 0.001, ** *p* < 0.01 and * *p* < 0.05 were considered significant.

To determine the optimal in vivo dosage of RP-832c, mice were treated by subcutaneous injection of 5 and 10 mg/kg RP-832c for 21 days, beginning three days after the administration of a single bolus (2.5 U/kg) of BLM. Masson’s Trichrome staining was used to assess lung architecture and collagen deposition. Figure 2A demonstrates that the overall extent of fibrosis was significantly decreased in BLM-challenged RP-832c-treated mice. Higher resolution images demonstrate that the alveoli of BLM-challenged vehicle-treated mice contained high amounts of fibrotic and collagenous tissue, decreasing the spaces between the alveoli which were not present in the BLM-challenged 5 or 10 mg/kg RP-832c-treated mice (Figure 2A). This finding correlated with lower Ashcroft scores for both treatment groups (Figure 2D). H&E staining of lung parenchyma lesions from the BLM-challenged vehicle-treated mice tissue sections exhibited variably non-existent lung structures, large fibrotic masses (50% of the microscopic field) with only partial lung architecture preserved, and multifocal obliteration of the alveoli by fibrous masses. Moreover, BLM-challenged vehicle-treated mice exhibited eosinophilic, amorphous, and slightly vacuolated material [32], as well as thickened alveolar septae with eosinophils, macrophages, lymphocytes, and plasma cells (Figure 2B). In contrast, the BLM-challenged RP-832c-treated groups demonstrated minimal fibrous thickening of alveolar/bronchiolar walls, fewer distended blood vessels, and less cellularity. A moderate thickening of alveoli walls without obvious damage was also observed in these animals (Figure 2B). We further observed a significantly lower macrophage count in the BLM-challenged RP-832c-treated animals compared to the BLM-challenged vehicle-treated mice (*p*-value < 0.001) (Figure 2E). Comparatively, mice treated with 10 mg/kg RP-832c showed a stronger reduction in fibrosis and macrophage counts compared to those treated with 5 mg/kg RP-832c. Hence, 10 mg/kg was used as a standard treatment dose for the remaining experiments. Interestingly, we observed a similar trend in preventing BLM-induced fibrosis for mice treated with RP-832c using intranasal administration of the peptide. (Supplementary Figure S4).

**Figure 3:**
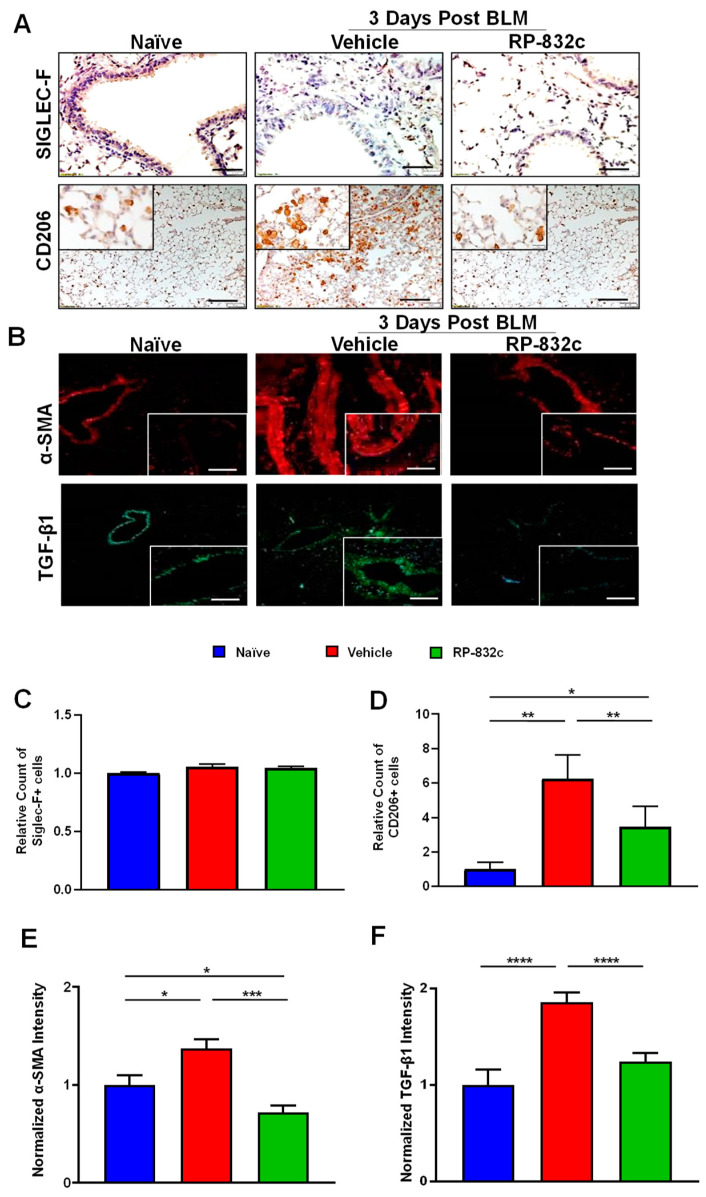
RP-832c peptide levels significantly decreased in CD206 + macrophages and the profibrotic markers α-SMA and TGF-β1 without affecting Siglec-F-positive alveolar macrophages. (**A**) Representative 20× and 40× images of immunohistochemistry staining using anti-Siglec-F and anti-CD206 antibodies in lung tissues of naïve mice, as well as BLM-challenged vehicle/RP-832c-treated mice. The calibration bar represents 10 µm. (**B**) Representative 20× and 40× images of immunofluorescence staining of lung tissues using antibodies for α-SMA (red) and TGFβ-1 (green). The calibration bar represents 20 µm. (**C**–**F**) Bar graphs showing the quantifications of the IHC (Siglec-F & CD206) and IF (α-SMA & TGF-β1) staining of lung tissues. Quantifications was done using Metamorph imaging software. S. E. **** *p* < 0.0001, *** *p* < 0.001, ** *p* < 0.01 and * *p* < 0.05 were considered significant.

CD206 expression was significantly decreased in BLM-challenged RP-832c-treated mice compared with BLM-challenged vehicle-treated mice (*p*-value < 0.001) (Figure 3A,D). Furthermore, we observed no significant difference in Siglec-F/CD170 (a marker for resident alveolar macrophages) expression levels between the different groups (Figure 3A,C). Because the role of TGF-β1 is well established in promoting fibrosis by inducing a fibroblast to myofibroblast switch, we further measured the fibrosis markers TGF-β1 and alpha-smooth muscle actin (α-SMA). BLM-challenged RP-832c-treated lung tissue showed a significant reduction (*p*-value < 0.001) in both α-SMA (Figure 3B,E) and TGF-β1 (Figure 3B,F) expression compared with BLM-challenged vehicle-treated mice.

### 3.3. RP-832c Peptide Treated Firosis and Reduced Expression of CD206 and Fibrosis Markers in a Late Model of BLM-Induced Lung Fibrosis Model

**Figure 4:**
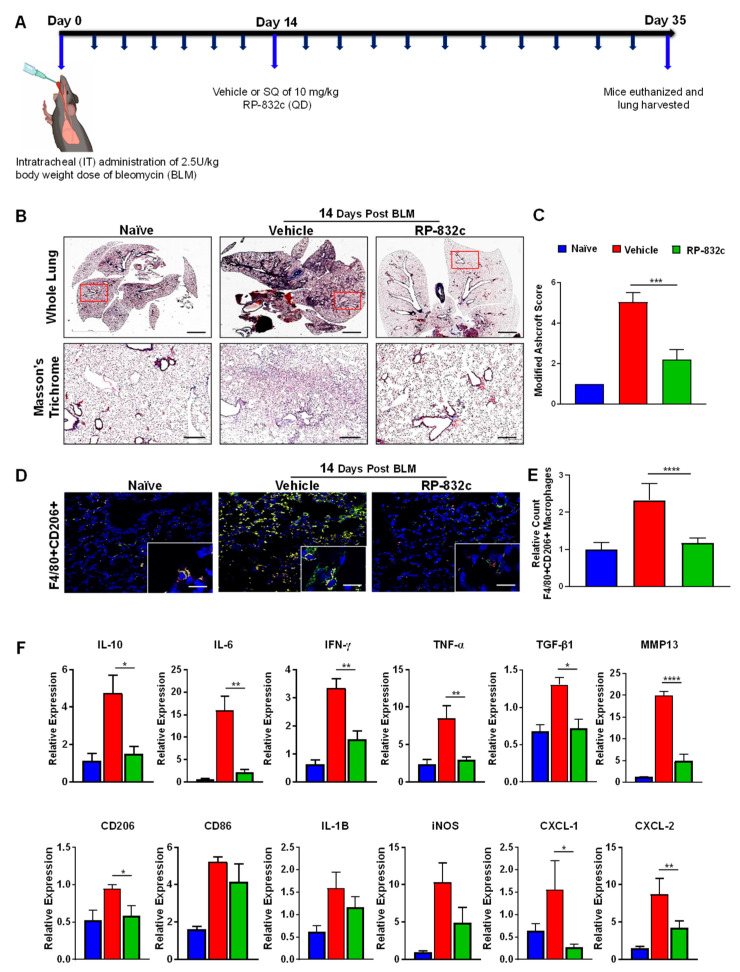
RP-832c treatment blocks the “cytokine storm” in an established late model of BLM-induced lung fibrosis. (**A**) A schematic of the animal study in which mice were challenged with 2.5 U/kg body weight dose of BLM on day 0. A total of 14 days post-BLM challenge, mice were treated with either a vehicle or 10 mg/kg RP-832c (QD) for an additional 21 days. (**B**) The upper panel shows 2× representative images of Masson’s Trichrome staining of whole lung tissue, and the lower panel contains representative 20× images of Masson’s Trichrome-stained lung tissue. (**C**) A bar graph for the modified Ashcroft scoring of the lung tissues. (**D**) Representative 20× and 40× images of immunofluorescence staining of lung tissue sections with anti-F4/80 (red) and Anti-CD206 (green). (**E**) Quantification of F4/80 & CD206 double-positive macrophages in the IF staining of the lung tissues. Quantification was performed by measuring counts in 10 separate fields using Metamorph imaging software. (**F**) RT-PCR of IL-8 functional homologs (CXCL-1, CXCL-2), inflammatory cytokine markers (CD206, CD86, IL-6, IL-10, IFN-γ, TNF-α, IL-1B, iNOS), and fibrosis markers (TGF-β, and MMP-13) using RNA extracted from FFPE tissue sections. S. E. **** *p* < 0.0001, *** *p* < 0.001, ** *p* < 0.01 and * *p* < 0.05 were considered significant.

To determine the effectiveness of RP-832c on established fibrosis, RP-832c treatment was initiated 14 days after the BLM treatment (Figure 4A). As expected, the RP-832c treatment significantly reduced the modified Ashcroft scores compared to the vehicle-treated group (Figure 4B). Furthermore, we observed a significant decrease in lung CD206 expression by immunofluorescence (Figure 4D). M2 macrophages are responsible for the upregulation of pro-inflammatory and pro-fibrotic cytokines in the lung, commonly known as the “cytokine storm” [33,34]. To determine whether RP-832c treatment affects these cytokine levels, we performed RT-PCR on mRNA extracted from FFPE tissues of the naïve, BLM-challenged vehicle-treated, and BLM-challenged RP-832c-treated tissue sections. Interestingly, we observed significant decreases in TNF-α, IL-10, IL-6, CXCL1, and CXCL2 expression among the RP-832c treatment groups (Figure 4E). We further observed decreases in M1 macrophage markers CD86, IL-1β, and iNOS expression, although they did not reach statistical significance (Figure 4E). Concomitantly, fibrosis markers TGF-β1 and MMP-13 were significantly decreased in RP-832c-treated mice (Figure 4E). Collectively, our findings demonstrated that a reduction in CD206-positive macrophages correlates with a significant reduction in inflammatory pro-fibrotic cytokines, which in turn reduces myofibroblasts and collagen deposition: the hallmarks of many types of fibrotic diseases (Graphical Abstract).

**Figure 5:**
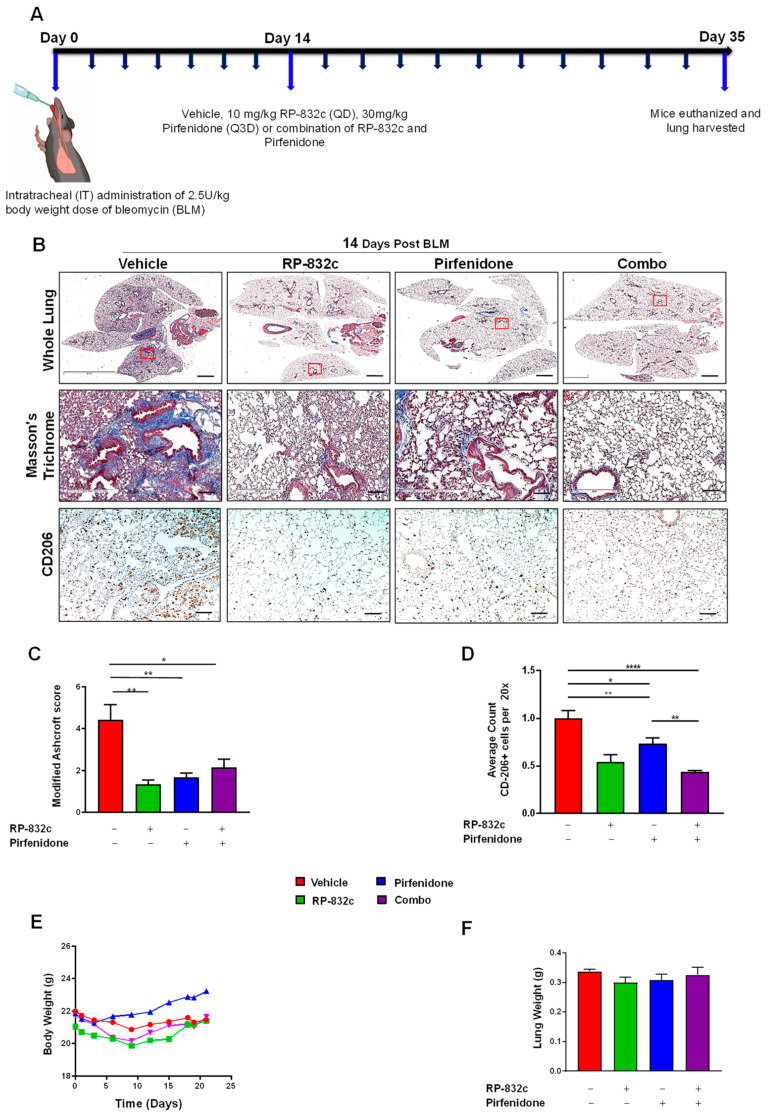
RP-832c peptide is comparable to Pirfenidone in decreasing fibrosis in an established late model of BLM-induced lung fibrosis. (**A**) A schematic of the animal study in which mice were challenged with a 2.5 U/kg body weight dose of BLM at day 0. A total of 14 days post-BLM challenge, mice were treated with either a vehicle, 10 mg/kg RP-832c (QD), 30 mg/kg Pirfenidone (Q3D), or a combination of RP-832c and Pirfenidone for an additional 21 days. (**B**) Histological analysis of lung tissues. The upper panel shows representative 2× images of Masson’s Trichrome-stained whole lung tissue, the middle panel contains representative 20× images, and the lower panel shows 10× images representing anti-CD206 immunohistochemical staining of lung tissue. (**C**) Modified Ashcroft scoring of each treatment group, determined after 21 days of treatment. *n* = 6 per treatment group. S. E. ** *p* < 0.001, and * *p* < 0.05 were considered significant. (**D**) Quantification of the anti-CD206 IHC-stained lung tissues. The bar represents 10 µm. *n* = 6 per treatment group. S. E. **** *p* < 0.0001, ** *p* < 0.001, and * *p* < 0.05 were considered significant. (**E**) Body weights of the mice measured over the course of treatment in each group. *n* = 6 per treatment group. (**F**) Lung weights of the mice measured at the end of the study. *n* = 6 per treatment group.

Pirfenidone and Nintedanib are the only two FDA-approved drugs for lung fibrosis [35,36]; therefore, we tested any synergistic effects of RP-832c peptide with these drugs. A daily treatment of 10 mg/kg of RP-832c was compared to 30 mg/kg Pirfenidone (Q3D) or 50 mg/kg Nintedanib (Q3D) treatments for 21 days, starting at 14 days post-BLM challenge. Combination therapy was also evaluated (Figure 5A and Figure S5). As a monotherapy, RP-832c demonstrated a similar modified Ashcroft score to Pirfenidone (Figure 5B). Interestingly, when we compared CD206 expression levels, RP-832c treatment showed significant decreases in CD206 as expected, while the combination of RP-832c with Pirfenidone showed a significant decrease compared to mice in the single treatment groups (Figure 5C). Although these differences were not statistically significant, there was a similar trend of complementary activity between RP-832c and Pirfenidone in decreasing tissue expression of TGF-β1 and α-SMA. Interestingly, RP-832c did not synergize with Nintedanib; however, RP-832c treatment inhibited fibrosis to a greater extent compared with Nintedanib as a monotherapy (Supplementary Figure S5).

### 3.4. RP-832c Lacks Significant Toxicity

To determine RP-832c-associated toxicity, mice treated with 50 mg/kg were assessed for body weights, organ weights, complete blood count, and blood chemistry. No significant differences were observed (Table 1 and Supplementary Figure S6A).

## 4. Discussion

It is evident from various studies that macrophages are indispensable in tissue repair and subsequent fibrotic changes. Macrophages demonstrate remarkable plasticity and can acquire M1 and M2-like phenotypes, which both drive and resolve fibroproliferative responses to injury. M1 macrophages initiate host defense responses against pathogens by generating reactive nitric oxide (NO) via inducible nitric oxide synthase (iNOS), and by releasing proinflammatory cytokines and chemokines such as IL-1β, IL-12, IL-23, CCL2, and TNF-α. Meanwhile, M2 macrophages contribute to the pathogenesis of fibrosis by secreting anti-inflammatory and profibrotic growth factors such as TGF-β1 [37]. To modulate these M2 macrophages, we previously reported the development of first in-class HDP-derived peptides, RP-182 and RP-832c, which specifically target the CD206 receptor on M2 macrophages [29]. These peptides are designed to specifically target the CRD5 domain of the CD206 receptor on M2 macrophages, which induces rapid folding of the CD206 receptor, inducing apoptosis of the CD206 positive macrophages, as demonstrated in our in vitro studies [29]. This current report specifically focuses on RP-832c, which was selected based on its enhanced stability and increased half-life compared to RP-182. 

In vitro, RP-832c showed a dosage-dependent inhibition of cell viability of M2 macrophages with an IC50 of ~6 µM, while producing only minimal cytotoxicity in M1-polarized macrophages. Similar to our in vitro cytotoxicity studies, RP-832c treatment induced a significant decrease in CD206 + macrophages in mice when administered 3- and 14-days post-BLM challenge. Furthermore, RP-832c treatment significantly reduced fibrosis scores, suggesting that the targeting of elevated CD206 + M2 macrophages contributed to a decrease in lung fibrosis. In our 14-day BLM model of lung fibrosis, RP-832c also significantly decreased the mRNA expression levels of multiple cytokines, including TNF-α, IL-6, IL-10, and IFN-γ in the lung. Because TNF-α induces neutrophil migration, we further observed significant decreases in CXCL1 and CXCL2 in RP-832c-treated mice. While each of these markers appeared to return with time to naïve mice levels, the M1 markers iNOS, CD86, and IL-1β remained higher than in naïve mice. This was not surprising, as we previously reported that RP-182 induces rapid repolarization of M2 macrophages to the M1 phenotype and triggers apoptosis in a subpopulation of CD206-positive macrophages [29]. This increase in M1 macrophages and decrease in CD206-positive M2 macrophages with either RP peptide treatment or in CD206 KO mice is associated with an increase in CD8 + T cells [29]. The fact that the overall lung architecture of BLM-challenged RP-832c-treated mice was similar to the naïve lung further highlights the fibrosis-resolving effect of the RP-832c peptide.

A critical factor in macrophage-associated lung fibrosis is the origin of M2 macrophages. In the normal injury-repair response, macrophages readily acquire an M2 phenotype, which promotes fibroproliferation [38]. These M2-polarized macrophages promote collagen synthesis and deposition, as well as remodeling of the extracellular matrix that contributes to the buildup of fibrotic tissue [18]. It is well characterized that CD206 is expressed on both tissue-resident alveolar macrophages and infiltrating monocyte-derived alveolar macrophages [39]. Our findings demonstrate that RP-832c did not influence the expression of Siglec-F/CD170, a marker for resident alveolar macrophages in the lung, suggesting that RP-832c targets activated CD206-positive macrophages derived from infiltrating monocytes, which have been suggested to contribute to fibrosis. There are two lines of evidence to support our findings. First, Misharin et al., demonstrated that in BLM mouse models of lung fibrosis, the deletion of monocyte-derived macrophages after their recruitment to the lung markedly attenuated the severity of fibrosis, whereas the deletion of tissue-resident alveolar macrophages did not affect fibrosis severity [8]. Second, the depletion of circulating monocytes in CCR2−/− mice or via the administration of liposomal clodronate reduced fibrosis severity, implicating monocyte-derived cells in the development of fibrosis [40]. We did not observe any significant effect on the proliferation of multiple fibroblast cell lines cultured in vitro. Thus, it appears that RP-832c treatment specifically affects monocyte-derived M2-polarized activated macrophages, without reducing the protective resident alveolar macrophages.

Because infiltrating macrophages are critically responsible for contributing to fibrosis, as seen in IPF, several reports have demonstrated that the production of profibrotic cytokines such as TGF-β1 and IL-4 polarizes monocytes toward the M2-like phenotype, thus upregulating CD206 expression [41,42]. CD206 + M2-like macrophages in turn secrete cytokines such as TGF-β1 that promote fibroblast to myofibroblast transition [43]. Interestingly, RP-832c treatment of BLM-challenged mice decreased the thickness of the alveolar/bronchiolar wall. RP-832c also reduced collagen deposition and fibrosis by 30%, which is comparable with the effect of directly blocking TGF-β1 [44], which is a well-known mediator of converting fibroblasts to myofibroblasts and promoting fibrosis. Multiple reports demonstrate that the CD206 receptor is critical for ECM remodeling by macrophages and fibroblasts through the upregulation of MMPs, specifically MMPs 7, 11, and 13, which promote collagen turnover [45,46]. Indeed, we observed significant decreases in MMP-13 expression following the RP-832c treatment, as well as decreases in TGF-β1 and α-SMA tissue expression, which correlated to a reduction in the myofibroblast cells responsible for excessive collagen deposition and tissue remodeling observed in pulmonary fibrosis. Therefore, it is likely that the reversal of the macrophage-fibroblast crosstalk is responsible for the reduction in overall fibrosis observed with RP-832c treatment.

## 5. Conclusions

This research demonstrated that targeting CD206 + macrophages by HDP RP-832c showed a potential therapeutic effect in treating pulmonary fibrosis in mouse models. The significant effect of RP-832c on fibrosis appears to be through the targeting of M2 macrophages, as we did not observe direct inhibitory effects on the fibroblast cell lines. Currently, Nintedanib and Pirfenidone are the only approved compounds for lung fibrosis treatment [47]. Although the mechanism of action of Pirfenidone is not known, we observed decreased CD206 expression when both RP-832c and Pirfenidone were administered as monotherapies, with a trend toward significance in the combined treatments. This further suggests that peptide-based pulmonary fibrosis therapeutics may confer synergistic effects with current FDA-approved pulmonary fibrosis therapeutics. Although TGF-β1 antibodies have been proposed as an anti-fibrosis treatments, they have not fared well in clinical settings. Targeting of the CD206 receptor on M2 macrophages with RP-832c can serve to inhibit the cellular source of TGF-β1, with effects comparable to targeting TGF-β1 directly, but with significantly less toxicity.

In summary, HDPs offer the potential for significant anti-fibrotic activity, with the advantage of no observed toxicity at five times the therapeutic inhibitory dosages. Immunotherapies are now being proposed as treatment options for patients with IPF. RP-832c appears to fit in this class of immunotherapies, and could serve as a potential option for treating pulmonary fibrotic illnesses.

## 6. Patents

Patent No.: US 10, 149, 886 B2 was issued to Riptide Bioscience, Inc.

## Figures and Tables

**Table 1:** RP-832c peptides do not significantly affect whole blood cell counts and blood chemistry.

	Saline	RP-832c 50 mg/kg		
Parameter	*n* = 5	*n* = 10	Mean Difference	*p* Value
RBC/mm^3^	8.3 ± 0.6	8.5 ± 0.7	0.2	0.7295
WBC/μL	5.7 ± 1.8	7.7 ± 3.2	2.0	0.2163
Total Protein (g/dL)	5.6 ± 0.8	5.9 ± 0.5	0.3	0.5797
AST (mg/dL)	140.0 ± 75.5	91.2 ± 21.7	−48.8	0.1237
Phosphorus (mg/dL)	8.8 ± 2.4	7.7 ± 0.5	−1.1	0.2623
Creatinine (mg/dL)	0.2 ± 0	0.2 ± 0	0.0	-
ALT (mg/dL)	35.8 ± 18.9	27.2 ± 10	−8.6	0.3808
Albumin (g/dL)	2.9 ± 0.3	3.1± 0.3	0.2	0.3982
BUN (mg/dL)	22.4 ± 3.4	24.0 ± 4.3	1.6	0.3087
Globulin (g/dL)	2.6 ± 0.5	2.7 ± 0.2	0.1	0.4413
Alb/Globulin Ratio	1.1 ± 0.2	1.2 ± 0.1	0.1	0.3295
BUN/Creatinine Ratio	111.9 ± 17	120.1 ± 21.6	8.2	0.3087

## Data Availability

The data presented in this study are available on request from the corresponding author.

## Supplementary Materials

The following supplementary materials are available online at https://www.mdpi.com/article/10.3390/cells12091254/s1: Figure S1: Dose Response Curve of BMDM and validation of in vitro BMDM polarization assay. Figure S2: RP-832c shows no cytotoxicity against lung fibroblasts. Figure S3: CD206 expression is increased by BLM treatment. Figure S4: Intranasal administration of RP-832c peptide shows similar activity to subcutaneous administration. Figure S5: RP-832c peptide decreased fibrosis to a greater extent compared to Nintedanib. Figure S6: RP-832c peptide lacks toxicity.
