# Supplementary material for: A Novel CD206 Targeting Peptide Inhibits Bleomycin-Induced Pulmonary Fibrosis in Mice
Source: Cells. 2023 Apr 26;12(9):1254. doi: 10.3390/cells12091254 (PMC10177262; doi:10.3390/cells12091254)
Supplement: Supplementary file 1 [file cells-12-01254-s001.zip › RP832c in IPF supplemental figures Revised Version 04082023.pptx]

## Slide 1
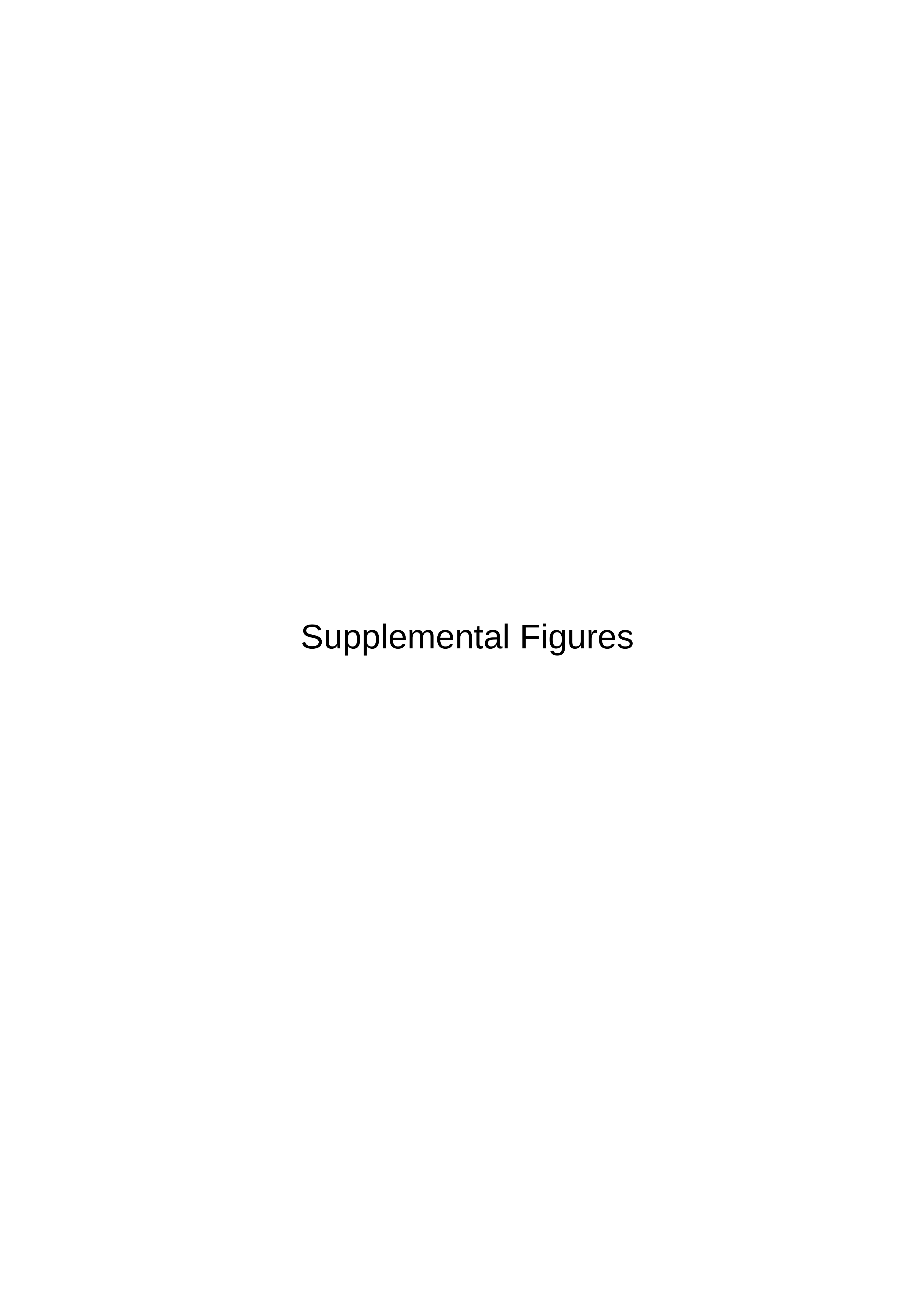

Supplemental Figures

## Slide 2
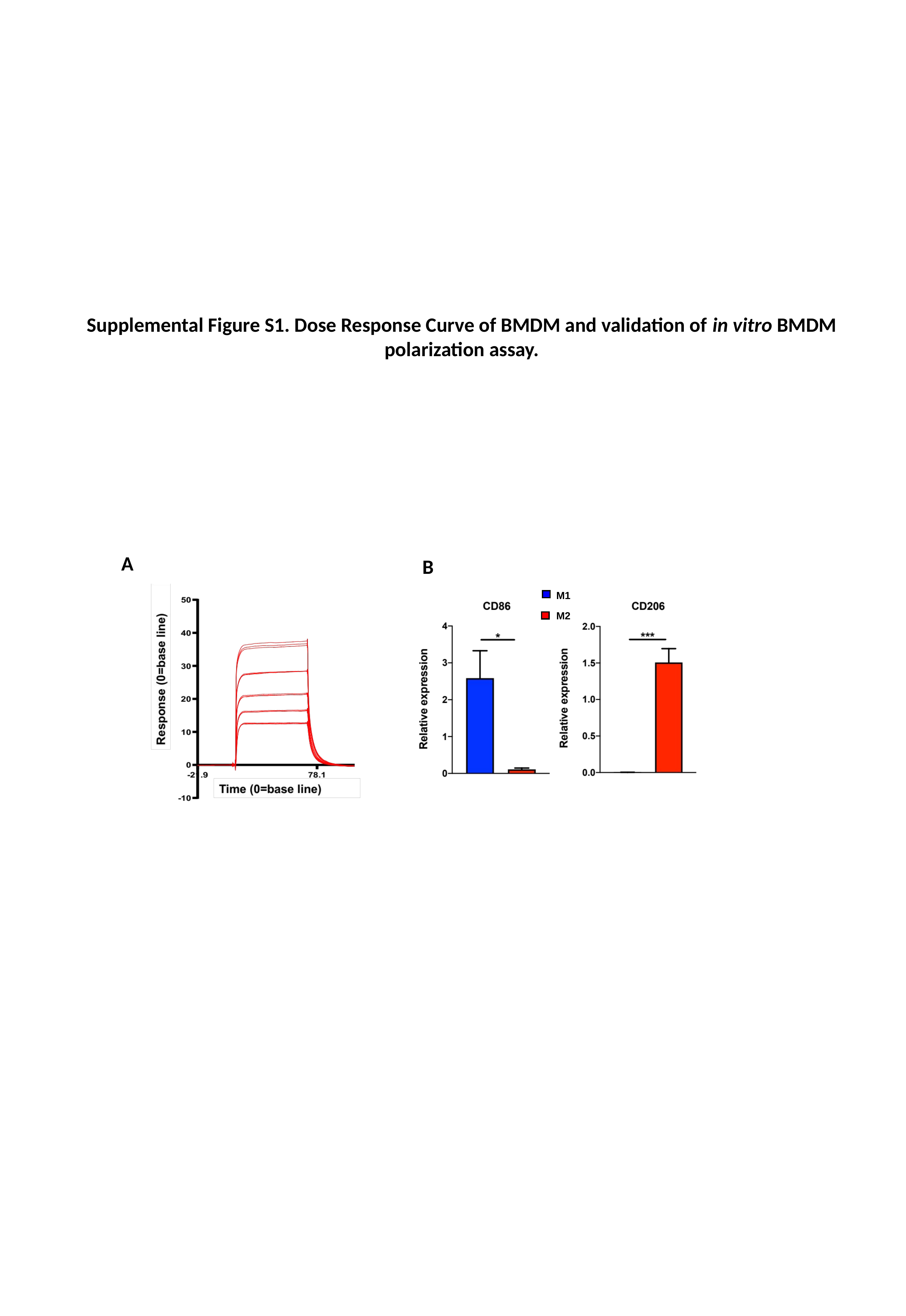

Supplemental Figure S1. Dose Response Curve of BMDM and validation of in vitro BMDM polarization assay.
A
B
M1
M2

## Slide 3
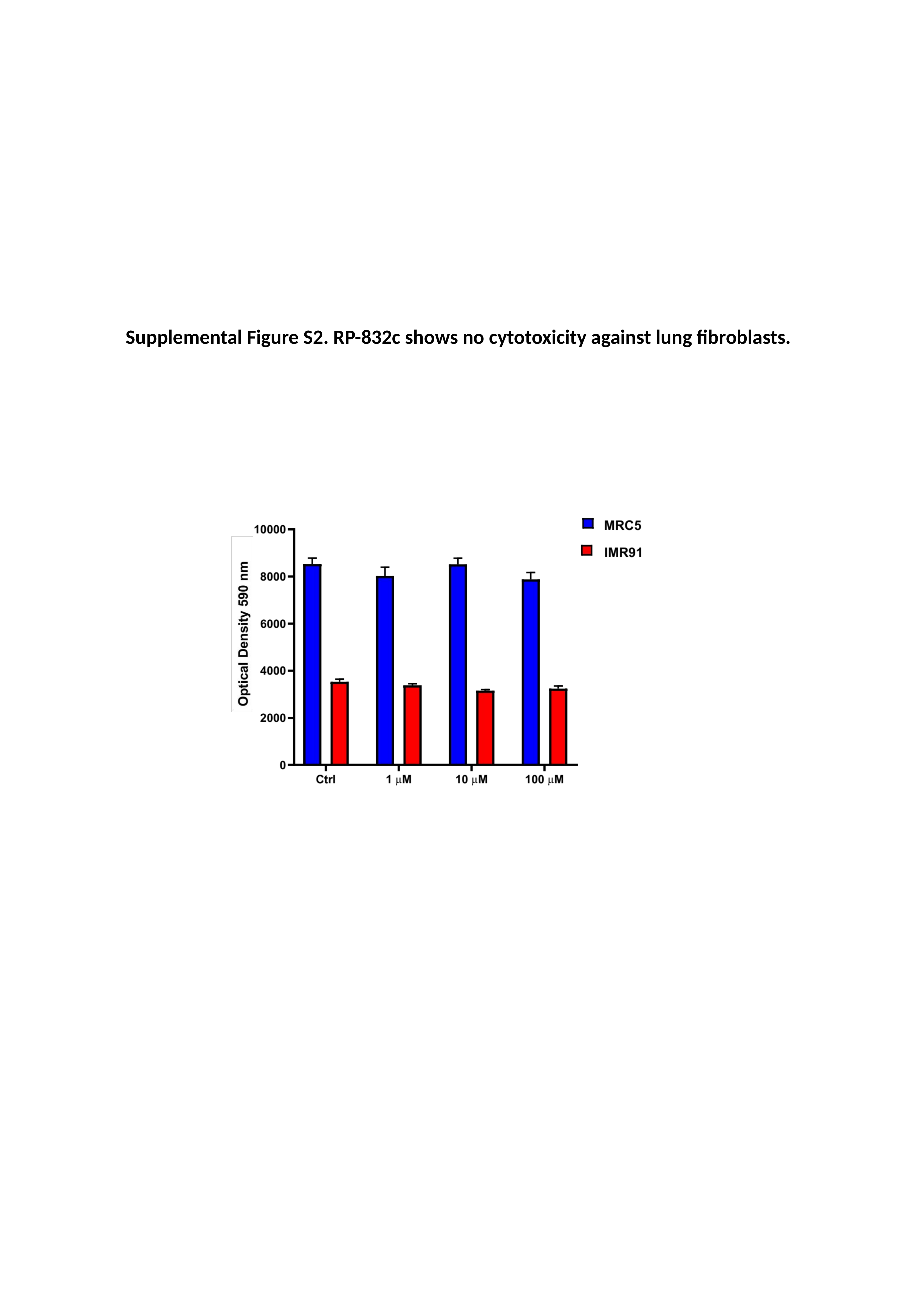

Supplemental Figure S2. RP-832c shows no cytotoxicity against lung fibroblasts.

## Slide 4
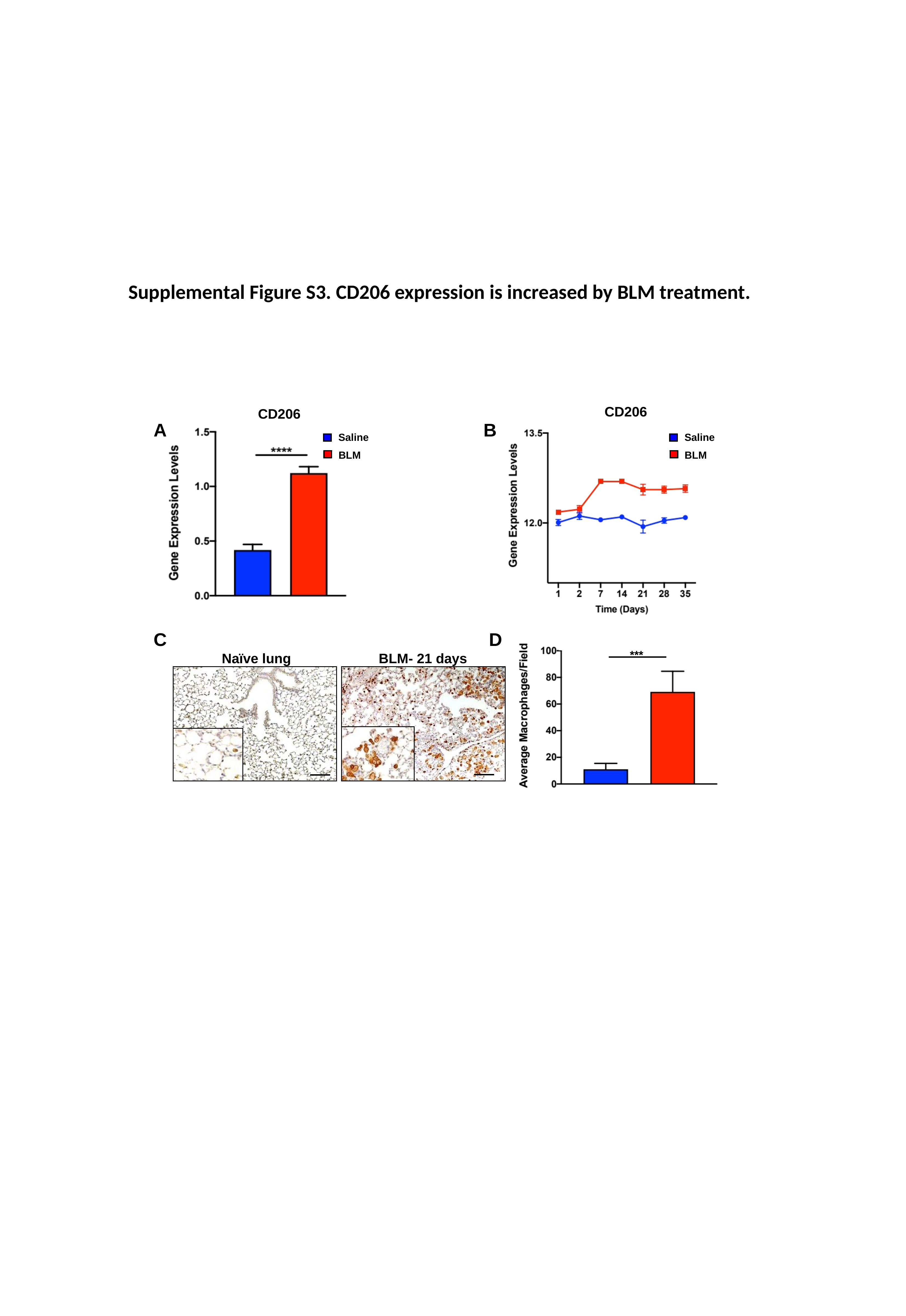

Supplemental Figure S3. CD206 expression is increased by BLM treatment.
CD206
CD206
A
B
Saline
BLM
Saline
BLM
C
D
 ***
BLM- 21 days
Naïve lung

## Slide 5
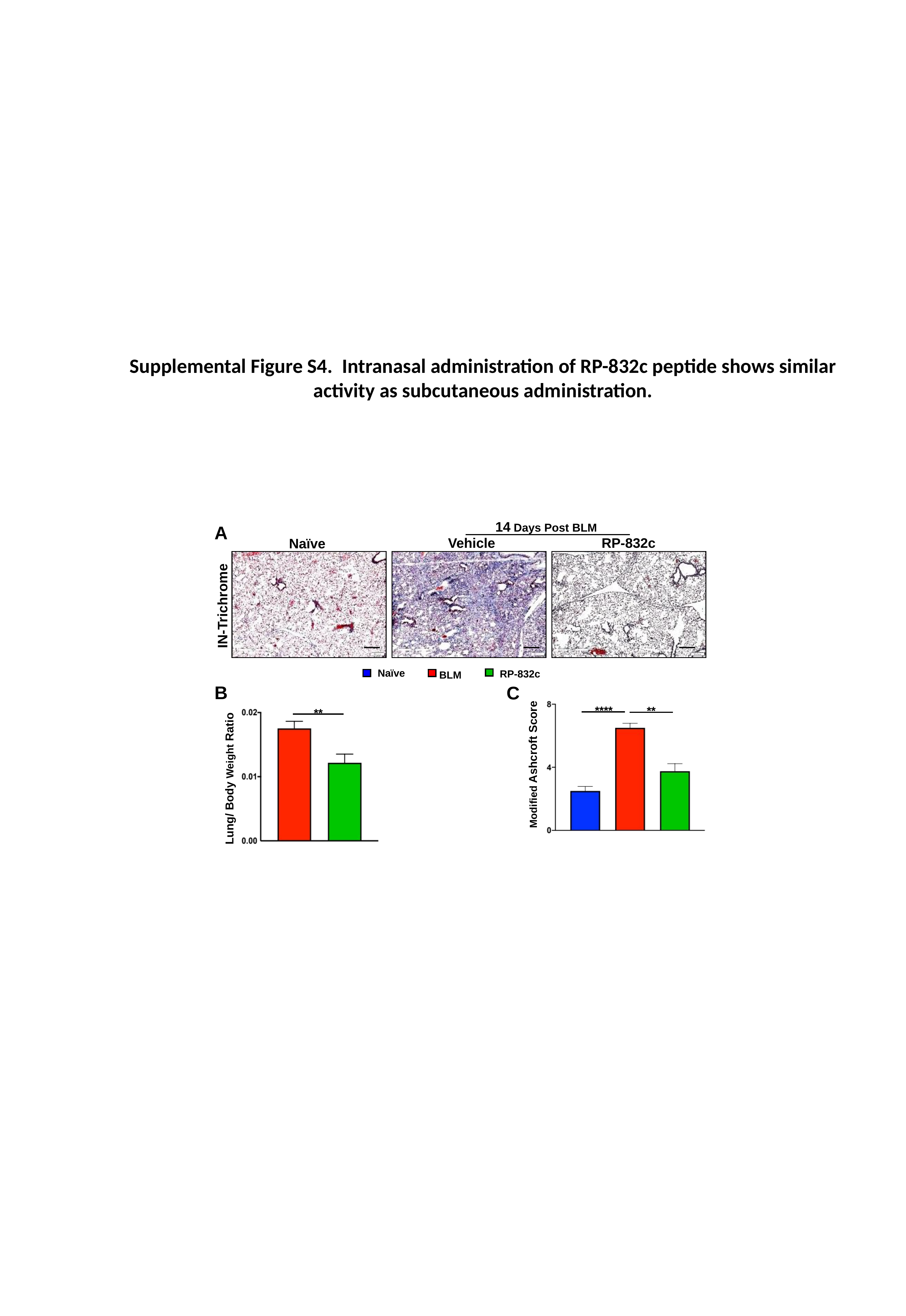

Supplemental Figure S4. Intranasal administration of RP-832c peptide shows similar activity as subcutaneous administration.
14 Days Post BLM
A
Naïve
IN-Trichrome
RP-832c
BLM
Naïve
B
C
 ****
**
**
Modified Ashcroft Score
Lung/ Body Weight Ratio
 Vehicle
RP-832c

## Slide 6
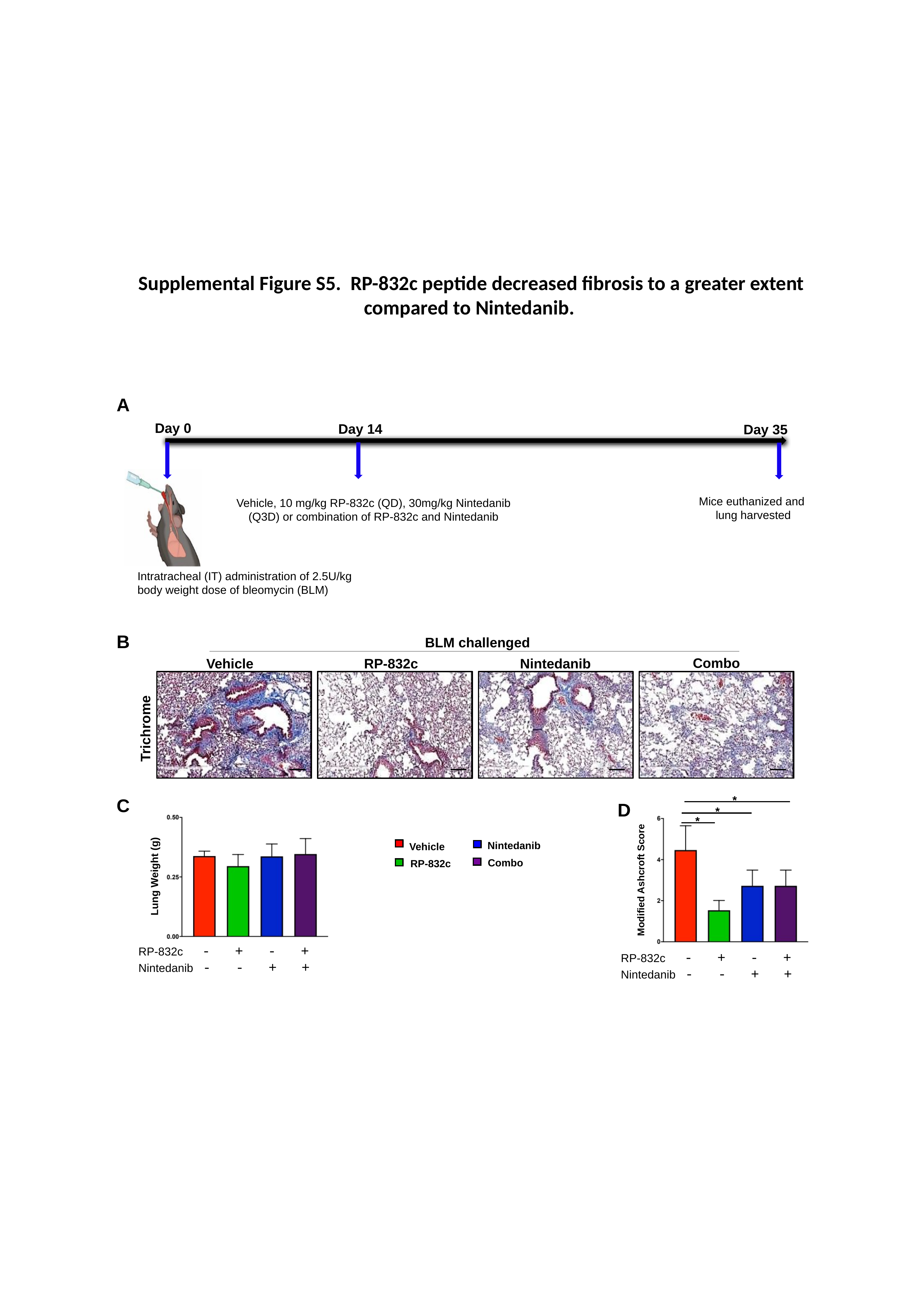

Supplemental Figure S5. RP-832c peptide decreased fibrosis to a greater extent compared to Nintedanib.
A
Day 0
Day 14
Day 35
Mice euthanized and
 lung harvested
Vehicle, 10 mg/kg RP-832c (QD), 30mg/kg Nintedanib (Q3D) or combination of RP-832c and Nintedanib
Intratracheal (IT) administration of 2.5U/kg body weight dose of bleomycin (BLM)
B
Combo
RP-832c
Vehicle
Nintedanib
Trichrome
 *
 *
 *
C
D
Modified Ashcroft Score
Lung Weight (g)
Nintedanib
 Vehicle
Combo
RP-832c
RP-832c - + - +
Nintedanib - - + +
BLM challenged
RP-832c - + - +
Nintedanib - - + +

## Slide 7
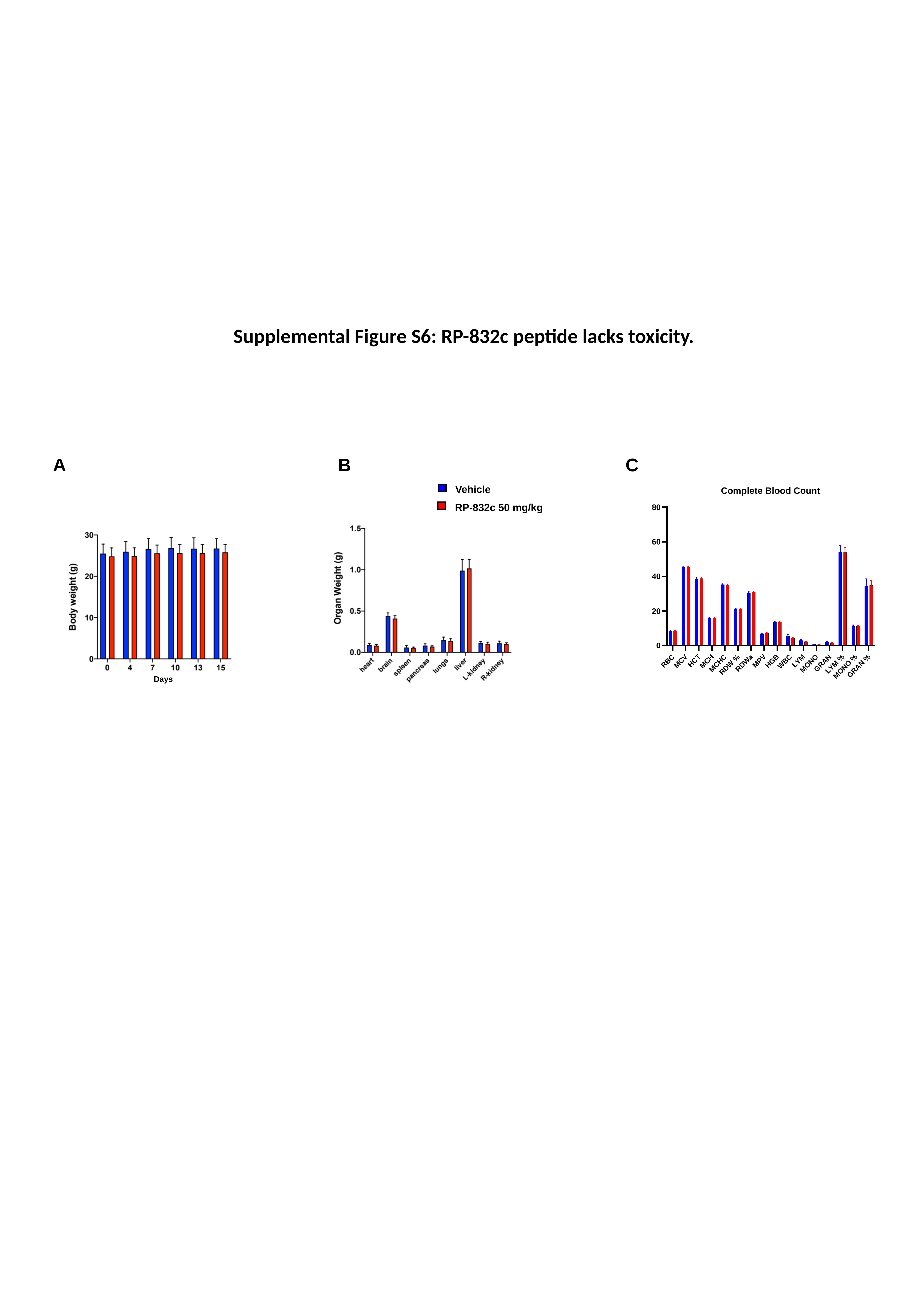

Supplemental Figure S6: RP-832c peptide lacks toxicity.
C
A
B
Vehicle
RP-832c 50 mg/kg
Days
