# Supplementary material for: A Novel CD206 Targeting Peptide Inhibits Bleomycin-Induced Pulmonary Fibrosis in Mice
Source: Cells. 2023 Apr 26;12(9):1254. doi: 10.3390/cells12091254 (PMC10177262; doi:10.3390/cells12091254)
Supplement: Supplementary file 1 [file cells-12-01254-s001.zip › RP832c in IPF Supplementary Figures Legend 04082023.pdf]

Supplemental Figures

Supplemental Figure S1. Dose-Response Curve of BMDM and validation of *in vitro* BMDM polarization assay. A. SPR (Surface Plasmon Resonance) dose response for the binding of CD206 receptor by different concentrations (0μM - 100μM) of RP-832c. **B.** Validation of *in vitro* BMDM polarization assay using C57BL/6 mice-derived macrophages. Bone marrow-derived macrophages polarized to M1 macrophages with 20ng/ml of M-CSF & 20ng/ml of INF-γ showed increased CD86 expression, while those polarized to an M2 phenotype using 20ng/ml of M-CSF & 20ng/ml of IL4 stimulation showed increased CD206 expression.

Supplemental Figure S2. RP-832c shows no cytotoxicity against lung fibroblasts. RP-832c (1μM - 100μM) did not show cytotoxicity against lung fibroblast cell lines MRC5 and IMR91. All data presented are the means of three independent experiments, performed in triplicate ± S. E. ***P < 0.0001, and ** P<0.001 and *P<.05 is significant.

Supplemental Figure S3. Analysis of publicly available data sets showed that CD206 expression is increased by BLM treatment. A. Secondary analysis of the GSE48455 data set (n=72) showed upregulation of CD206 expression in the BLM-treated mice compared to normal saline-treated mice. **B.** Secondary analysis of the GSE40151 data set (n=111) treated with BLM or normal saline for 35 days showed upregulation of CD206 gene expression with time. **C.** 10x and 40x IHC images representing anti-CD206 staining of lung tissues of BLM-treated mice compared to naïve mice lung tissues. **D.** Quantification of the IHC staining of CD206 of the lung tissues of BLM-challenged mice compared to lung tissues of naïve mice. *n=3* mice ***P < 0.0001, and ** P<0.001 and *P<.05 is significant.

Supplemental Figure S4. Intranasal administration of RP-832c peptide shows similar activity to subcutaneous administration. A. RP-832c peptide was administered intranasally at 25μg/mouse QD through day 24. Images are 10x representative images of Masson's Trichrome staining of each of the treatment groups. **B.** Lung-to-body weight ratio of lung tissues of mice from the different treatment groups. **C.** Modified Ashcroft score of lung tissues from the different treatment groups. ***P < 0.0001, and ** P<0.001 and *P<.05 is significant.

Supplemental Figure S5. RP-832c peptide decreased fibrosis to a greater extent compared to Nintedanib in an established late model of BLM-induced lung fibrosis model. A. A schematic of the animal study in which on day 0 mice were challenged with 2.5U/kg body weight dose of BLM; starting 14 days post-BLM challenge, mice were treated daily with 10mg/kg of RP-832c (QD), 30mg/kg Nintedanib (Q3D), or a combination of both for an additional 21 days. **B.** Representative 10x images of Masson's Trichrome staining show a significant reduction of collagen deposition in the lungs of RP-832c treated mice compared to the other treatment groups. **C.** Lung weights of mice from the different treatment groups were measured at the end of the study. **D.** Modified Ashcroft scoring of lung tissue sections from the different treatment groups. *n=6* per treatment group. S. E. ***P < 0.0001, and ** P<0.001 and *P<.05 is significant.

Supplemental Figure S6. RP-832c peptide lacks toxicity. A. Animal toxicity study conducted using CD1 mice showed that 50mg/kg RP-832c treatment did not have any significant effect on body weight compared to the vehicle-treated mice. **B.** Bar graph showing that 50 mg/kg of RP-832c treatment lacks any significant effect on the weight of various organs when compared to the vehicle-treated mice. **C.** Complete Blood Cell count shows that 50 mg/kg of RP-832c does not significantly affect the complete blood cell count compared to vehicle-treated mice.
